# Expanding access to near-vision correction services: WHO targets, tools, and initiatives

**Published:** 2026-02-19

**Authors:** Mitasha Yu, Stuart Keel

**Affiliations:** 1 Consultant: Vision and Eye Care Programme, Department of Noncommunicable Diseases, Rehabilitation and Disability, World Health Organization, Geneva, Switzerland.; 2 Technical Officer: Vision and Eye Care Programme, Department of Noncommunicable Diseases, Rehabilitation and Disability, World Health Organization, Geneva, Switzerland.

**Millions of people can regain their near vision if they are given access to spectacles for presbyopia. Here is what the World Health Organization recommends.**

**Figure:**
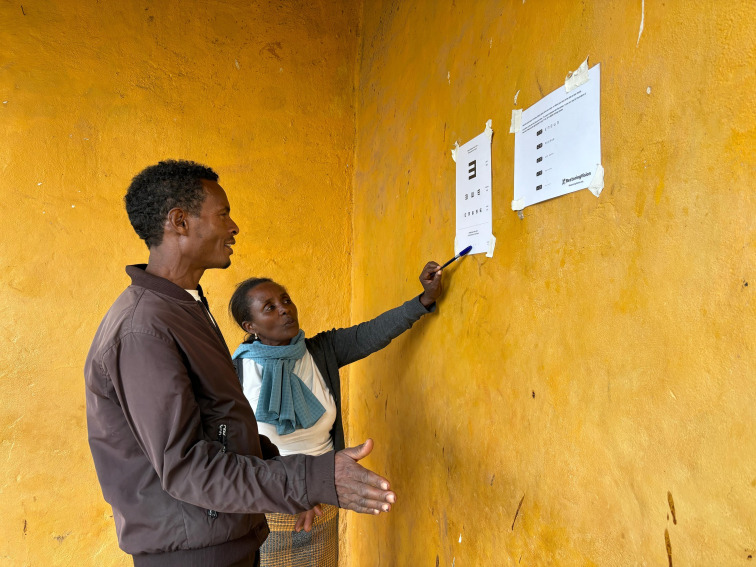
A community health worker checks visual acuity before dispensing near-vision spectacles. ETHIOPIA

© LAST MILE HEALTH CC BY-NC-SA 4.0

In 2021, the World Health Assembly endorsed the very first global target for effective refractive error coverage (see panel). The target recognises the major impact of near-vision impairment on daily life and productivity^1,2^ and includes spectacle coverage for both distance refractive error and near-vision impairment in older adults (presbyopia).

## Reaching the 2030 global target: what are the challenges?

Despite presbyopia being so common, access to affordable and good-quality near-vision spectacles remains limited. Current policies and workforce models in many countries often prevent over-the-counter provision of near-vision spectacles, and/or prevent community and primary health workers from dispensing these spectacles, even when adequate training and supervision could enable them to do so safely. In rural and remote areas, supply chains tend to be weak and spectacles are often unavailable. Cultural barriers also play a role, with many people viewing presbyopia as a natural part of ageing. In addition, there is a scarcity of data on effective refractive error coverage for near vision, making it difficult to monitor progress and undertake evidence-based planning.

## Models for the provision of near-vision spectacles

Spectacles are a simple solution to address presbyopia. Evidence shows that, for the majority of adults, ready-made near-vision spectacles are both effective and safe, and their simplicity means that most people can benefit immediately, without the need for complex equipment or highly specialised personnel.^3^ To meet the substantial need in low- and middle-income countries, it is crucial to make ready-made near-vision spectacles accessible as close as possible to people's homes.^4^

While countries can take many approaches to improving access, two key steps with the greatest potential impact are highlighted below.

### 1. Over-the-counter access

Over-the-counter provision of ready-made near-vision spectacles is one of the simplest and most effective ways to address presbyopia.^5,6^ In many high-income countries, including Australia, the USA, and the UK, provision of these spectacles without a regulated prescription has been permitted for decades. They are considered low-risk medical devices and are designed for safe self-selection, with evidence showing that most users can choose the correct power and that incorrect choices pose no health risk. Successful models from countries such as India, Brazil, Sweden, and South Africa demonstrate the feasibility of this approach.

Key considerations when planning for over-the-counter access in low- and middle-income countries include the following.

- National strategies should align over-the-counter provision with broader health system planning in eye care, including the workforce, delivery models, and supply chains. In high-income settings, over-the-counter near-vision spectacles are supported by readily available comprehensive eye care services. Replicating this in low-resource settings requires careful planning to ensure referral pathways are in place for individuals whose vision does not improve with these devices.
- A broader range of accessible providers, such as pharmacists and trained retail personnel, can support over-the-counter provision by offering vision and eye health screening and helping people choose appropriate strengths, supported by clear user instructions and health education materials that promote safe self-selection and indicate when further eye examination is needed.
- Safeguards are needed to ensure product quality standards are met, ensuring that ready-made near-vision spectacles are both safe and effective.
- Affordability, and the long-term sustainability of supplying good-quality ready-made near-vision spectacles, are essential so that they remain reliably available.
- Equity considerations are also critical. Women, people in rural areas, and marginalised groups often face the greatest barriers to access.
- Raising community awareness about near-vision impairment and the availability of ready-made near-vision spectacles is essential, helping to create and sustain demand for services.

### 2. Integration within primary and community health care

When integrating eye care within primary and community health settings, WHO recommends that, at a minimum, distance and near-vision screening and an external eye health screen is undertaken. If the distance-vision and eye health screen are passed, and only the near-vision screen is failed, near-vision spectacles can be trialled and dispensed. If there are any issues with their distance vision or eye health, or if near-vision spectacles do not improve their near vision, the person should be referred for a full eye examination. The full protocol is given in the **WHO Vision and eye health screening implementation handbook**.^7^ WHO notes that community health workers and primary health workers, as part of an integrated, competency-based refractive error team, can be safely trained to screen for near-vision impairment and provide ready-made near-vision spectacles.^8,9^ It is also important to address any concerns from eye care personnel who may feel uncertain about non-specialised workers, such as community and primary health workers, taking on these tasks. Clear communication can help show that this approach supports the whole team, improves access to care, and allows eye care personnel to focus on patients with more complex eye care needs.

“Presbyopia is universal, predictable, and correctable. The solutions are simple, scalable, and affordable.”

## Technical resources and normative work

To support countries addressing refractive error, including presbyopia, WHO has developed a range of products and tools:

- The WHO **Summary guides on quality standards for spectacles** (bit.ly/4rf2hip**)** help governments and key stakeholders to procure safe and effective products.
- The WHO **Eye care competency framework** (bit.ly/483EzHL), and the **Competency-based refractive error teams** resource (bit.ly/3Xet7CT), show that community health. workers and primary care workers can be safely trained to screen and dispense for presbyopia.
- The WHO **Refractive error situational analysis tool** (bit.ly/4oXnsP1) supports countries to integrate presbyopia into national eye care strategies.
- The WHO **Learning on TAP** (bit.ly/4rqkYcu) resource, and the **Vision and eye screening implementation handbook** (bit.ly/49FemAs), make training in vision and eye screening, and dispensing ready-made near-vision spectacles, widely accessible.
- The **WHOeyes app** (bit.ly/4ohzeBu) helps raise awareness about presbyopia and supports efficient screening when paired with an eye health screen.

## Conclusion

Presbyopia is universal, predictable, and correctable. The solutions are simple, scalable, and affordable. Achieving equity in access requires supportive policies, trained personnel, reliable supply chains, and strong partnerships. Provision of near-vision spectacles is an integral part of the WHO SPECS 2030 initiative. If countries adopt inclusive policies, millions of people could regain their near vision, thus unlocking opportunities for a better quality of life and economic prosperity.

**Global target for effective refractive error coverage (eREC)**

**Target.** A 40 percentage point increase in **eREC** by 2030. Countries with a baseline effective coverage rate of 60.0% or higher should strive for universal coverage.

**Definition of eREC.** The proportion of people in need of refractive error services who have received services (i.e. spectacles, contact lenses, or refractive surgery) and have a resultant good-quality outcome, relative to the number of people in need of refractive error services.

- For **distance vision**, a ‘good quality outcome’ is defined as presenting visual acuity (PVA) of ≥ 6/12 (known as ‘distance eREC’)
- For **near vision**, a ‘good quality outcome’ is defined as PVA ≥ N6 (known as ‘near eREC’)

**Definition of presenting visual acuity (PVA).** If spectacles or contact lenses are worn to the assessment, visual acuity is measured with the person wearing them.

**Relevant WHO initiatives and resources**

**SPECS 2030 initiative:** At a strategic level, presbyopia is embedded in the **WHO SPECS 2030 initiative** (bit.ly/4ohy2hu), which provides a global framework for improving refractive error coverage. Within this, presbyopia is seen as a potential ‘quick win’ to help accelerate progress toward the global eREC targets. This is because ready-made near-vision spectacles have lower product costs, require only basic workforce competencies, and can be supplied through simpler procurement processes using standard strengths that can be stocked in bulk. This creates a major opportunity to integrate services within community and primary care settings.
